# Optimized heart rate for 320-row cardiac CT can be feasibly predicted from prescan parameters

**DOI:** 10.1186/s40064-015-1478-5

**Published:** 2015-11-12

**Authors:** Eriko Maeda, Nobuo Tomizawa, Kodai Yamamoto, Shigeaki Kanno, Masaaki Akahane, Kenji Ino, Masae Uehara, Aiko Sakamoto, Toshiaki Semboku, Rumiko Torigoe, Kuni Ohtomo

**Affiliations:** Department of Radiology, Graduate School of Medicine, University of Tokyo Bunkyo-ku, 7-3-1 Hongo, Tokyo, 113-8655 Japan; Department of Radiology, New Tokyo Hospital, 1271 Wanagaya, Matsudo, Chiba 270-2232 Japan; Department of Radiology, Teikyo University School of Medicine Hospital, Mizonokuchi, 3-8-3 Mizonokuchi, Takatsu-ku, Kawasaki, Kanagawa 213-8507 Japan; Department of Radiology, NTT Medical Center Tokyo, 5-9-22 Higashi-Gotanda, Shinagawa-ku, Tokyo, 141-8625 Japan; Imaging Center, The University of Tokyo Hospital, 7-3-1 Hongo, Bunkyo-ku, Tokyo, 113-8655 Japan; Department of Cardiology, Graduate School of Medicine, University of Tokyo, 7-3-1 Hongo, Bunkyo-ku, Tokyo, 113-8655 Japan; Chronos Medical Device Incorporated Masago, 4-1-6, Mihama-ku, Chiba, 261-0011 Japan; Toshiba Medical Systems Corporation, Tokyo Metropolitan Regional Office, 1-6, Tsukuda 2-Chome, Chuo-ku, Tokyo, 104-0051 Japan

**Keywords:** Heart rate, 320-detector row CT, Breath hold, Contrast material

## Abstract

To evaluate the degree of heart rate (HR) changes at rest (HRrest), during breath hold (HRtest), and during cardiac CT examinations (HRscan) in a large group of patients , and to derive and asses the feasibility of a predictive formula for HRscan. HRrest, HRtest, and HRscan were retrospectively compared in a total of 563 consecutive patients who underwent 320-row cardiac CT. Multiple regression analysis was performed to derive predictive formulae for HRscan in the entire study population and, in each group of patients with decreased (Dec) or increased (Inc) HR during breath hold. The predictive formula was evaluated as accurate when less than 5 % of the actual HRscan exceeded the predicted HRscan by ±5 beats per minute (bpm). The average values of the HRtest (65.3 ± 12.0 bpm) and HRscan (63.7 ± 11.9 bpm) significantly decreased from those of the HRrest (68.4 ± 11.9 bpm) (p < 0.0001). The predictive formula (HRscan = 3.601 + 0.113HRrest + 0.8HRtest) was determined to be accurate only in Group Dec. The HRtest significantly decreased from the HRrest, and the HRscan significantly decreased from the HRtest. An accurate predictive formula for HRscan could be built only for Group Dec.

## Background

Cardiac computed tomography (CT) has long been performed with single-source, 64-row scanners or less. Scanning the whole heart requires 6–10 heartbeats, or even more when scanners have fewer rows of detectors. With the advent of 320- and 256-detector row CT and dual-source 64-row CT with Flash mode, it has become possible to perform most adult cardiac CT scans in 1–3 heartbeats. Using 320-row CT, patients with heart rates (HR) lower than 75 beats per minute (bpm) can be scanned with one beat, with some adjustments in their acquisition windows based on the HR. In particular, the acquisition window should be widened to scan the systolic phase as well as the diastolic phase, when the HR is predicted to be relatively fast. In contrast, when the HR during the actual scan is lower than expected, the patient would be exposed to unnecessary radiation during systolic scanning. In this regard, prediction of the scan HR is necessary for patients with low HR, particularly if between 65 and 75 bpm. For high HR patients, multi-segment reconstruction may be used to improve temporal resolution (Tomizawa et al. 2013). In multi-segment reconstruction, accurate prediction of HR is necessary to determine the number of scans (i.e., heartbeats) and the time of the gantry rotation, both of which affect temporal resolution (Halliburton et al. 2003; Herzog et al. 2007). Therefore, HR prediction before cardiac CT scan is crucial for both low and high HR patients.

Past studies on changes in HR during breath holding and contrast injection during cardiac CT are small, and gave controversial results. None of these studies involved 320-row cardiac CT (Zhang et al. 2008; Horiguchi et al. 2011; Christensen et al. 2011; Becker et al. 2011). We hypothesized that scan HR (HRscan) can be predicted from pre-scan parameters, such as HR at rest (HRrest) and during breath hold (HRtest).

## Methods

### Patients

This study was approved by the institutional review board, and was performed in accordance with the ethical standards laid down in the 1964 Declaration of Helsinki and its later amendments. The requirement for informed consent to participate in this study was waived, because of the retrospective design. The patients’ records and information were made anonymous before starting the analysis.

The records of 791 consecutive patients (470 men, 321 women; mean age 65.5 ± 13.9 years, range 8–94 years) who underwent cardiac CT angiography from August 2011 to July 2013 were retrospectively reviewed. The patients were suspected of having coronary artery disease, had a history of myocardial infarction, or had a complex cardiac anomaly. The exclusion criteria were arrhythmia, such as atrial fibrillation and flutter (n = 43), premature ventricular contraction during HR recording (n = 16), premature atrial contraction during HR recording (n = 4), complete left bundle branch block (n = 4), complete right bundle branch block (n = 1), proxysmal supraventricular tachycardia (n = 1), sick sinus syndrome (n = 1), and sustained ventricular tachycardia (n = 1); presence of pacemakers (n = 7); wide-volume scanning in patients who underwent coronary artery bypass surgery (n = 88); expiratory scanning for ablation planning (n = 9); irregular protocol for concurrent evaluation of the pulmonary artery or right ventricle (n = 8); pediatric patients (n = 2); and inaccurate ECG-recording (n = 43).

The final study group included 563 patients (311 men, 252 women; mean age 65.2 ± 12.9 years, age range 18–94 years; body weight 61.3 ± 14.4 kg, range 32–142 kg). Medications that could possibly influence HRs were beta blockers in 290 patients (51.5 %), digitalis derivatives in 110 patients (19.5 %), Calcium channel blockers in 86 patients (15.3 %), nifedipine in 21 patients (3.7 %), anti-arrhythmcs in seven patients (1.2 %), and alpha-blockers in three patients (0.5 %).

### CT data acquisition

All examinations were performed by 320-detector CT scanner (Aquilion ONE Vision Edition: Toshiba, Tochigi, Japan) with prospective ECG gating axial scans.

The scanning parameters were as follows: detector configuration, 320 × 0.5 mm; gantry rotation time, 275, 300, 320 or 350 ms depending on breath hold HR; tube potential, 120 kV; and tube current, from at 250 to 760 mA depending on body habitus. Acquisition window and number of scans were determined based on the experience of attending radiologists (EM, NT, KY, and SK). Patients received 22.2 mgI/kg of Iopamidol 370 mgI/mL (Iopamiron 370: Bayer, Osaka, Japan); the mean volume administered was 45.7 ± 10.4 mL (range, 25–96 mL) over 14 s. Bolus tracking was performed using thresholds of 100 (HU) in the left ventricle and 260HU in the descending aorta. Patients were assigned to breathe in and hold their breaths after the first threshold. The scan was immediately started after the second threshold.

As a baseline medication, oral β-blocker was administered to 116 patients. For 173 outpatients with HRs higher than 75 bpm, 20–40 mg of metoprolol (Lopresor: Novartis, Tokyo, Japan), was administered. The patients were instructed to take the medicines 2 h prior to the examination. In 2013, we started to use an intravenous β-blocker, landiolol at 0.125 mg/kg (Corebeta; Ono Pharmaceutical, Osaka, Japan) for patients with HRs higher than 75 bpm. Eighteen patients received injection before the test breath hold, and were scanned 4–7 min after injection. Before 2013, no additional β-blocker was used when the HR was higher than 75 bpm at the time of the examination. No patients who were administered β-blockers had contraindications, such as hypotension, more than grade II atrioventricular block, severe pulmonary hypertension causing right-sided cardiac failure, severe cardiac failure, and allergy to β-blockers. There were no side effects from β-blockers recorded. All patients received 2.5 mg sublingual isosorbide dinitrate (Nitorol; Eisai, Tokyo, Japan) before imaging. No nitrates were administered to patients with contraindications, such as severe hypotension, closed angle glaucoma, and allergy to nitrates.

### Acquisition of heart rate

**Fig. 1:**
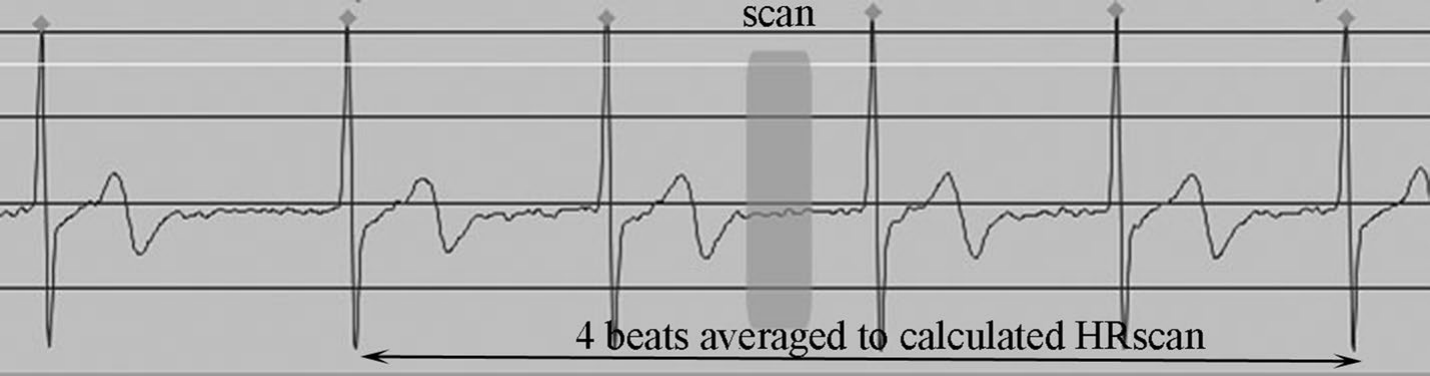
Definition of the four beats and the timing of exposure. HRscan was defined as the average HR of consecutive four beats (*arrow*), with the second beat corresponding to the first CT cardio angiogram scan. The scan duration took place during the RR interval within the *gray box*. The beginning of the scan was the left edge of the *gray box*, and the end of the scan was the right edge of the *gray box*

HR was recorded in terms of RR interval on ECG (IVY Model 3000; Chronos, Chiba, Japan). Mean HR during free breathing for 10 s (HRrest) was immediately recorded prior to giving instructions for the test breath hold; the actual test breath hold lasted for 10 s. HRtest was defined as the average HR of four beats, with the first beat designated as the one occurring at 5 s of the test breath hold. HRscan was defined as the average HR of four consecutive beats, with the second beat corresponding to the first CT cardio angiogram scan (Fig. 1).

### Statistical analysis

All statistical analyses were performed using JMP Pro software (version 10.0.2; SAS, Cary, NC). Quantitative variables were expressed as mean ± standard deviation.

The differences in HRrest, HRtest, and HRscan were determined by the Bland–Altman method and were analyzed in the entire population as well as in the groups of patients in whom the HRtest decreased from the HRrest (Group Dec) and those in whom the HR test increased from the HRrest (Group Inc). This analysis based on groups was performed to determine whether the behavior of HR during breath hold was related to the behavior of HR after contrast injection; for this purpose, multiple regression analysis was performed after extracting patients in whom the HRtest decreased from the HRrest. The ratios of changes in HRscan in both groups were analyzed using Fisher’s exact test.

Multiple regression analysis was based on data acquired during odd-numbered months. The derived multiple regression predictive formula was then applied to data acquired during even-numbered months, to assess accuracy of prediction. This analysis was performed on the entire study population, Group Dec, but not on Group Inc because the number during the even-numbered months (n = 36) was too small to calculate the 5 % assessment of feasibility. The prediction was evaluated as accurate when less than 5 % of the actual HRscan exceeded the predicted HRscan by ±5 bpm; this criterion was based on the fact an error of ±5 bpm will result in inadequate 1-beat scan or unnecessary 2-beat scan when the predicted HR was 70–75 bpm. A *p* value of <0.05 was determined as significant.

The accurate formula was applied to the HRrest and HRtest to calculate the estimated HRscan. A theoretical acquisition window based on the predictive formula and percentage of RR interval was determined from the estimated HRscan and was compared with the actual acquisition window. If the actual acquisition window was longer than the theoretical acquisition window, the examination was determined to have an excessive acquisition window. The percentage of examinations with excessive acquisition window in the concerned group was estimated.

Clinical parameters, such as sex, age, weight, height, body mass index, amount of contrast material, speed, β-blockers used, history of diabetes mellitus, and standard deviation of HR at rest were compared between the Groups Dec and Inc. The same parameters were also compared between the patients whose HRscan decreased from HRtest vs HRscan increased from HRtest, in Group Dec and Group Inc respectively. For comparison, Student’s t test was applied for quantitative parameters and Fisher’s exact test was applied for categorical data. The significance level was adjusted by Bonferroni to 0.05/10 = 0.005.

## Results

**Fig. 2:**
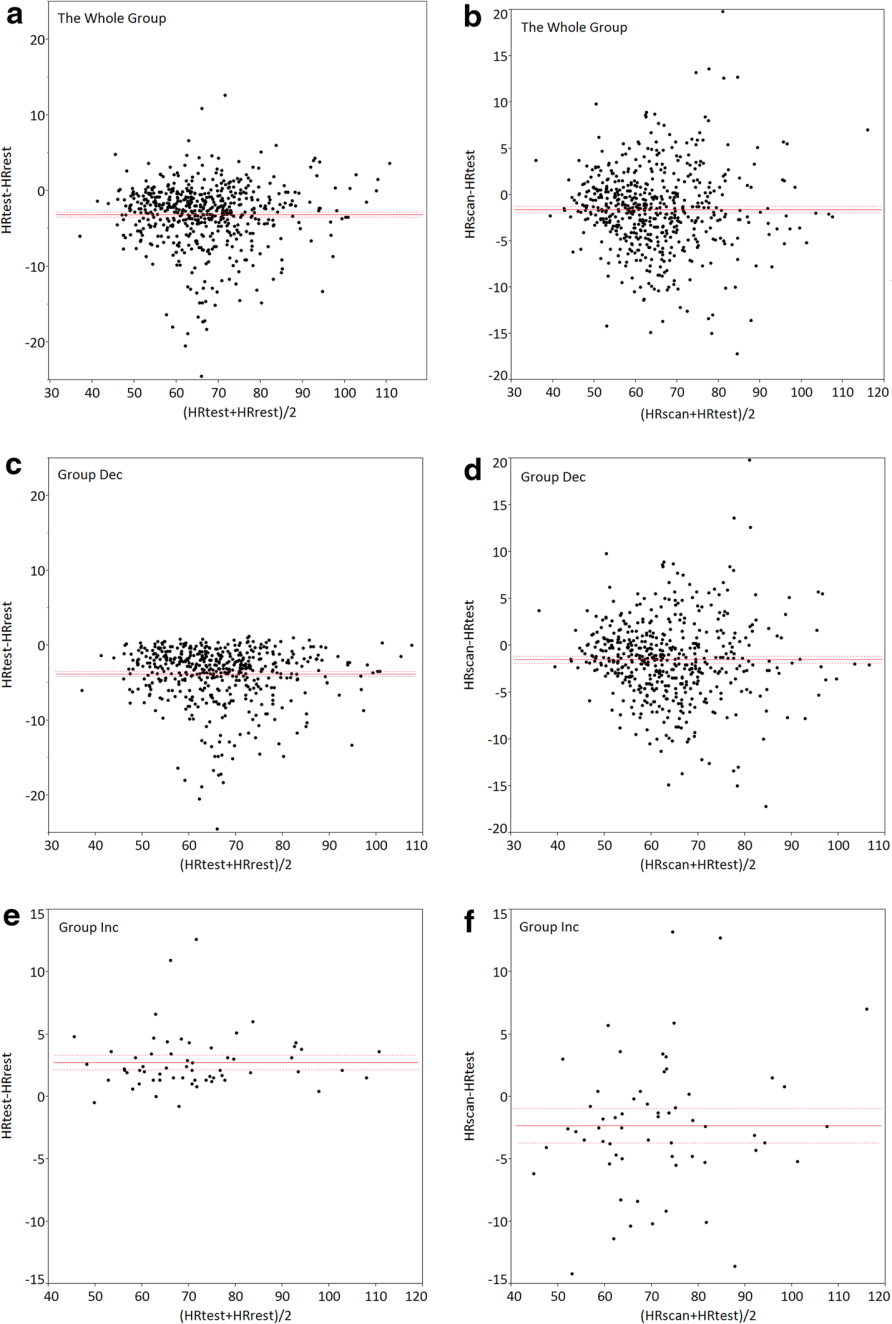
Bland–Altman plot of HRrest versus HRtest and HRtest versus HRscan in the entire study population (**a**, **b**); In Group Dec (**c**, **d**); and in Group Inc (**e**, **f**). The *solid grey line* indicates bias. The two *broken gray lines* indicate limits of agreement

The measured HRrest, HRtest, and HRscan are shown in Table 1 and Fig. 2. HRtest significantly decreased by approximately 3 bpm from the HRrest; the HRscan significantly decreased by approximately 1.5 bpm from the HRtest. The average duration between the end of the breath hold instruction and the actual scan was 4.3 s. Bland–Altman analysis revealed statistically significant differences between HRrest and HRtest and HRtest and HRscan in all groups. The behavior of HR in Groups Dec and Inc are shown in Table 2. The ratios of changes in the HRscan in Groups Dec and Inc were not significantly different from each other (p = 0.83).

**Table 1:** Comparison of the HRrest, HRtest, and HRscan among the three groups

	HRrest (bpm)	HRtest (bpm)	HRscan (bpm)	HRrest versus HRtest (bpm)		HRtest versus HRscan (bpm)	
				Bias	Limits of agreement	Bias	Limits of agreement
Overall	68.4 ± 11.9	65.3 ± 12.	63.7 ± 11.9	−3.11*	−2.77 to −3.46	−1.60*	−1.24 to −1.95
HRtest decreasing group	68.3 ± 11.6	64.4 ± 11.3	62.9 ± 11.2	−3.81*	−3.48 to −4.14	−1.51*	−1.14 to −1.88
HRtest increasing group	69.9 ± 14.3	72.7 ± 14.4	70.4 ± 15.3	2.75*	2.16 to 3.33	−2.33*	−0.95 to −3.70

*HR* heart rate, *BPM* beats per minute

* Statistically significant

**Table 2:** Classification of patients according to HRchanges during examination

	HRtest		
	Decrease from HRrest (= Group Dec)	Increase from HRrest (= Group Inc)	Total
HRscan			
Decrease from HRtest	388 (77.1)	47 (78.3)	435
Increase from HRtest	115 (22.9)	13 (21.7)	228
Total	503	60	563

Data are presented as number (%)

*HR* heart rate

The results of multiple regression analysis are shown in Table 3. For the overall study population, the predictive formula based on data during odd-numbered months (n = 293) was calculated as follows:

$${\text{HRscan}} = 3.368 + 0.096\;{\text{HRtest}} + 0.823\;{\text{HRtest}}$$

**Table 3:** Results of multiple regression analysis

Population	Factor	Coefficient (β)	Standard error	t value	p value
Overall (n = 293)	HRrest	0.096	0.063	1.514	0.013*
	HRrest	0.823	0.063	13.05	<0.001*
Group Dec (n = 273)	HRrest	0.113	0.068	1.670	0.0095*
	HRrest	0.800	0.069	11.58	<0.001*

*HR* heart rate

*Statistical significance p < 0.05

Applying this formula to the data during even-numberd months (n = 270), the actual HRscan was out of the ±5 bpm range in 57 patients (19.5 %), indicating that the formula reached our criteria for accuracy. For the Group Dec, the predictive formula based on data during odd-numbered months (n = 273) was calculated as follows:

$${\text{HRscan}}\;{ = }\; 3. 6 0 1\,{ + }\, 0. 1 1 3\;{\text{HRrest}}\,{ + }\, 0. 8\;{\text{HRtest}}$$

**Fig. 3:**
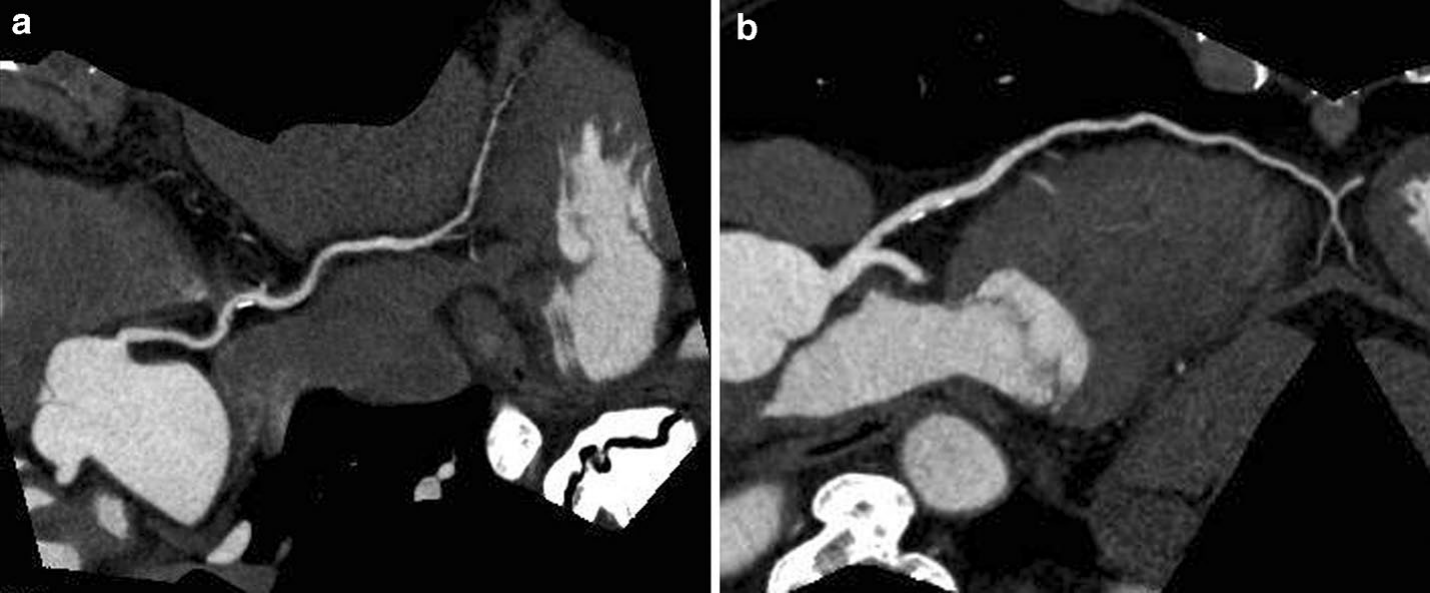
ECG-gated cardiac CT with curved multi-planar reconstruction in a 68-year-old man with multiple coronary risks. A rest HR of = 72 beats per minute was scanned with a narrow acquisition window and effective dose of 1.32 mSv at a HR of = 68 beats per minute to obtain high-quality images of the RCA (**a**) and LAD (**b**). HRrest, HRtest, HRscan were 72, 70, and 68 bpm, respectively. If HR was kept at 72 bpm throughout the examination, it would have been necessary to set a systole–diastole acquisition window (i.e. 30–80 %).* RCA* right coronary artery,* LAD* left anterior descending artery

Applying this formula to the data during even-numbered month (n = 232), the actual HRscan was out of ±5 bpm range in only four patients (1.5 %), indicating that the formula was likely to be feasible. This formula was applied to Group Dec and the estimated HRscan was calculated; the actual acquisition window was assessed as excessive in 80 patients (14.2 %) (Fig. 3).

No significant difference was observed in clinical parameters between Group Dec and Group Inc, and their subgroups (Table 4).

**Table 4:** Comparison of clinical parameters among groups of patients who underwent cardiac CT examinations

	Overall			Group Dec			Group Inc		
	Group Dec	Group Inc	p	HRscan Dec	HRscan Inc	p	HRscan Dec	HRscan Inc	p
Sex (M: F)	284:219	27:33	0.15	229:160	56:59	0.097	19:28	8:5	0.25
Age (years)	65.1 ± 12.9	63.7 ± 17.0	0.44	65.5 ± 12.3	63.6 ± 14.8	0.16	66.0 ± 13.9	55.2 ± 24.0	0.04
Body weight (kg)	61.4 ± 14.5	58.7 ± 15.0	0.18	61.0 ± 13.3	62.7 ± 17.8	0.28	59.1 ± 14.5	57.3 ± 17.4	0.7
Body height (m)	1.61 ± 0.10	1.59 ± 0.11	0.18	1.61 ± 0.10	1.59 ± 0.11	0.07	1.58 ± 0.11	1.63 ± 0.09	0.16
Body mass index	23.6 ± 4.2	23.2 ± 4.7	0.45	23.4 ± 3.82	24.5 ± 5.33	0.015	23.6 ± 4.6	21.5 ± 4.9	0.14
Contrast material (ml)	45.8 ± 10.4	44.2 ± 10.6	0.26	45.5 ± 10.1	47.1 ± 11.1	0.16	44.8 ± 11.1	41.2 ± 8.5	0.28
Injection speed (ml/s)	3.6 ± 0.7	3.4 ± 0.7	0.04	3.6 ± 0.7	3.6 ± 0.8	0.64	3.4 ± 0.6	3.2 ± 0.8	0.35
β blocker (−/+)	238/265	35/25	0.16	190/199	48/47	0.8	26/21	9/4	0.45
Diabetes mellitus (−/+)	336/167	44/16	0.41	258/131	78/37	0.81	34/13	10/3	0.8
SD of HR in rest	1.89 ± 4.27	2.14 ± 3.45	0.67	1.82 ± 4.59	2.13 ± 2.94	0.5	1.70 ± 2.40	3.70 ± 5.76	0.06

## Discussion

To the best of our knowledge, this is the first study on a large population of 563 patients that analyzed HR changes during cardiac 320-detector row CT. Our results revealed significant decreases in HR by breath hold and contrast injection. The HR test decreased from HR rest in approximately 90 % of patients, but increased from HRrest in 10 % of patients. HRscan significantly decreased from HRtest, regardless of the changes in HR during breath hold. For Group Dec, it was feasible to predict HRscan from HRrest and HRtest with the formula that we specified, with 14.2 % of the scans having an excessive acquisition window.

The RR interval is influenced by various factors, such as arterial pressure receptors, chemical receptors, cardiac receptors, and stretch receptors in the lungs and thoracic cavity (Wheeler and Watkins 1973; Horiguchi et al. 2006; Cooke et al. 1998; Clynes 1960; Angelone and Coulter 1964). Respiration is the most influential factor for the RR interval; upon inhalation, a negative intrathoracic pressure increases venous return and HR (Clynes 1960). However, although HR increases for 4 s, it is also known to decrease for 10 s afterwards; this may explain the behavior of HR during breath hold (Clynes 1960). Although the HRtest and HRscan in the present study were recorded to be approximately 4 s after the breath hold instruction, patients started holding their breaths after the inhalation instruction that came before the breath hold. Therefore, we consider that breath hold duration was actually longer than 4 s.

Decrease of the HRscan from the HRtest may be partially explained by deep inhalation during breath hold at scanning. One possible explanation for the HRscan being lower than the HRtest is the difference in frequency and depth of respiration, which are known to influence RR interval (Angelone and Coulter 1964). Fast contrast injection may also contribute to decrease in HR, but we could not find the supporting evidence.

The results of previous small-scale studies that determined HRscan decreasd from HRtest have been heterogeneous and controversial. Zhang studied 101 patients who underwent 64-row cardiac CT and concluded that HR decreased by approximately 4 bpm during breath hold, but was same during the scan and at rest (Zhang et al. 2008). Horiguchi analyzed 112 patients who underwent 64-row coronary CT and found that HR during coronary angiogram scan was almost the same as the HR during non-contrast calcium scoring scan obtained with breath hold (Horiguchi et al. 2011). They also found that HR range was wider during coronary angiogram scan than during calcium scoring scan; therefore. HR during coronary angiogram scan may be difficult to predict. Christensen studied 64-row cardiac CT in 60 patients and compare Iopamidol-370 versus Iodixanol-320 (Christensen et al. 2011). They found no significant HR changes between the breath hold HR and scan HR when Iopamidol-370 was used; whereas the scan HR significantly decreased by approximately 2 bpm from breath hold HR when Iodixanol-320 was injected, by about 2 bpm. Becker studied 64-row dual-source cardiac CT in 96 patients and compared Iomeprol-400 versus iso-osmolar Iodixanol-320; they concluded that there were no significant HR changes between the calcium scoring scan and coronary angiogram scan (Becker et al. 2011). The results of the present study on a large population shows that both breath hold and contrast injection were associated with significant decreases in HR during scanning, at least for 320-row cardiac CT.

The derived predictive formula was feasible for Group Dec and could retrospectively determine the examinations with theoretically excessive acquisition window. As a next step, we would like to prospectively apply this formula on patients with decreased HR during breath hold to predict the HR at scan and to determine the width of acquisition window. Further validation of the formula would be possible by comparing image qualities between experience-based and formula-based cardiac CT scans.

For Group Inc, we could not derive a predictive formula because the number of patients was too small to perform a feasibility study. As shown in Table 2, the HRscan increased from the HRtest in 21.7 % of patients in the Group In. This percentage of patients was prone to scan failure, particularly when HR was 65–75 bpm. Therefore, if a patient showed increase in HR during breath hold, it may be better to widen the acquisition window when the rest HR was higher than 65 bpm, and to increase the number of scans when the rest HR was higher than 75 bpm.

This study had some limitations. We could not analyze variation in HR in a longer span of time because the HRrest was recorded for only 10 s, whereas some of the past pulmonary or abdominal studies recorded the rest HR for 5 min (Chartrand-Lefebvre et al. 2011; Sahani et al. 2007; Romano et al. 2009). This restriction was due to the original setting of the ECG that we used. Another limitation was the likelihood that the timing of starting inhalation and breath hold varied from patient to patient. Many patients started to inhale at the beginning of instructions, but some started after the end of instructions for inhalation, or even after the breath hold instruction.

## Conclusions

The HRtest significantly decrease by approximately 3 bpm from the HRrest in 90 %, and the HRscan significantly decrease by approximately 1.5 bpm from the HRtest. Predictive formula for HRscan seems to be accurate and prevent excessive acquisition window for Group Dec patients.
